# A comparison of raspberry freezing-damage during preservation in isochoric and isobaric conditions

**DOI:** 10.3389/fnut.2024.1439726

**Published:** 2024-07-29

**Authors:** Ștefan Ioan Câmpean, George Andrei Beșchea, Beatrice Georgiana Vuțoiu, Maria Bianca Tăbăcaru, Gabriel Năstase

**Affiliations:** Department of Building Services, Faculty of Civil Engineering, Transilvania University of Brasov, Brasov, Romania

**Keywords:** *Rubus idaeus* L. (Raspberry), constant volume, preservation, weights loss, brix, pH, microscopy

## Abstract

**Introduction:**

Fruits are perishable, thus it’s crucial to have an efficient preservation technique that can increase storage time while keeping physical quality and nutritional attributes in order to avoid wastage. The majority of methods for long-term storage require refrigeration.

**Methods:**

In this investigation, we assess the viability of isochoric freezing as a different technique of raspberry (Rubus idaeus L.) preservation. Raspberries were subjected to different storage conditions: isochoric freezing at –4°C, conventional isobaric settings at +3°C (refrigerator), –21°C (freezer), and –4°C with a trehalose solution in a plastic bag. The study assessed changes in weight loss, visual appearance, color, hardness, °Brix values, and pH over a seven-day period.

**Results:**

Key findings reveal that raspberries subjected to isochoric freezing below the freezing point of water experienced minimal weight loss after seven days. Visual appearance, color, hardness, °Brix values, and pH were comparable to fresh raspberries, indicating minimal alteration.

**Discussion:**

These results suggest that isochoric freezing shows potential as a preservation method that maintains the physical and chemical properties of raspberries similar to fresh fruit. Implementing diverse preservation techniques tailored to raspberries may contribute to environmental sustainability by reducing food wastage and the associated environmental impact.

## 1 Introduction

Raspberries are considered the most common berries in the world (1) and are highly appreciated by consumers (2–4). They are etaerio fruits with favorable growing conditions in the specific climate of the northern hemisphere, which is the predominant place where they are consumed. They are closely related to blackberries and other brambles or cranberries (5). Raspberries are very perishable fruits due to their high-water content, and due to their high respiration and transpiration rates, these characteristics predispose them to crushing. The berry consumption CAGR (compound annual growth rate) is estimated at 3.5% starting from 2022 to 2027, with the biggest market in Asia-Pacific and the fastest-growing market in North America (6). In accordance with the Food and Agriculture Organization of the United Nations (FAO), the whole quantity of berry production (raspberries, blueberries, strawberries, cranberries, gooseberries, fruits of the genus Vaccinium n.e.c., and other berries) registered from 1961 to 2020 was 310.83 megatons (7). If we are looking only at the raspberries, this represents 23.0077 megatons, and this represents only 7.4% of the total production. In the top 10 raspberries producers in the world, we found seven countries from Europe (Russian Federation, Poland, Serbia, Spain, Ukraine, Portugal, Bosnia-Herzegovina) and three countries from the Americas (Mexico, United States, and Chile). The biggest producer on the planet is the Russian Federation, with 182,000 metric tons per year (8). Raspberries are a highly nutritious fruit, packed with vitamins, minerals, and antioxidants. Raspberries (*Rubus idaeus* L.) contribute to the human diet (9, 10). They are important sources of dietary fibers important for health, obtained from whole foods, containing 6.5 g per 100 g of raw weight and, on a diet basis, 12.5 g per 100 g of kcal (11). They also contain calcium (25 mg/100 g), iron (0.69 mg/100 g), magnesium (22 mg/100 g), phosphorus (29 mg/100 g), potassium (151 mg/100 g), vitamin C (total ascorbic acid; 26.2 mg/100 g), and vitamin B-6 (0.055 mg/100 g), which makes them important in protecting human health (12). Also, studies have shown that extracts from red strawberries slow tumor progression in breast cancer cells (13) and have antioxidant, anti-inflammatory, and cell-protecting effects (14). A simple Google search for “raspberry preservation” returns more than 26 million results. If we add one more keyword to the search, “fresh,” then the new result number will decrease to around 7 million results. According to the huge number of results, we consider that there is a general interest in trying to store the raspberries for longer periods. The most common methods to preserve them are to dry them (15), freeze them (16) or make jam from them (17). All the most commonly used methods affect the fruit’s characteristics, such as texture (18) or physicochemical properties (19). Recent research has generally focused on enhancing the traditional and well-known preservation techniques for fruits (20, 21). Accordingly, we believe there is a significant opportunity to find innovative methods of preserving raspberries.

Isobaric freezing is a quick and common method for preserving fruits and vegetables both at home and on a large scale. The freezing method affects quality and nutritional value. Damage mechanisms include physical, chemical, and microbiological reactions during freezing, mechanical damages from freezing water in tissues, and ongoing chemical reactions post-harvest that cause deterioration. Freezing fruits at their peak ripeness is crucial, as enzymes in fruits can cause nutritional loss, color and flavor changes, and mass changes; these enzymes can be inactivated by freezing to prevent chemical reactions. Most fruits, containing 90% water, experience cell wall rupture due to ice crystals forming during freezing, leading to irreversible damage to turgor, firmness, and water holding capacity, especially in fragile fruits like cherries, blueberries, and raspberries. This results in suboptimal thawed products with reduced consumer acceptance. Innovative freezing techniques are being explored to reduce energy expenditure and improve frozen food quality. One of these emerging technologies involves the utilization of high-pressure isochoric freezing (22). Diversifying the preservation methods of fruits for longer periods of time can overcome geographical distribution and encourage productivity. Freezing fruits is an environmental stress, but by using the right method, these problems can be avoided. Fresh raspberries need to be consumed in 1 to 3 days if stored in the refrigerator. After this time, they can be stored in the freezer for at least 6 months at atmospheric pressure (isobaric) and temperatures below the freezing point of water.

The potential of fruit preservation by isochoric freezing has already been evaluated for cherries (22), tomatoes (23), and pomegranate (24) and the advantages of long-term food storage in the frozen state by isochoric concept have been evaluated in the preliminary analysis of Năstase et al. (25). According to the findings in isochoric preservation, it appears feasible and secure to conserve biological material in isochoric chambers at temperatures as low as −4°C while maintaining the composition of the preservation medium and minimizing the potential for kinetic ice nucleation caused by external disturbances (26, 27). Our findings indicate that the energy necessary for the preservation of a fixed amount of biological material in an isochoric freezer at −5°C is approximately one-third of the energy required for preservation in a conventional isobaric freezer at 5°C. Moreover, as per reference (28), the superiority of food quality preserved in an isochoric system is observed in comparison to that preserved in an isobaric system. The research objective is to compare the effects of isochoric and isobaric freezing on raspberry preservation, specifically focusing on the quality, nutritional value, and structural integrity of the fruit post-thaw. This study aims to investigate how different freezing methods impact physical and chemical damage mechanisms and overall consumer acceptance, with the goal of identifying techniques that minimize energy expenditure and enhance the quality of frozen raspberries.

## 2 Materials and methods

### 2.1 Fruit material

Fresh raspberries (2–5 g) were obtained from a local producer (Pades Berries SRL, Timisoara, RO) in Romania. The samples were stored at +3°C until further sample processing. Samples were used for experiments 2 days after harvest. All the raspberries used in these experiments were in the same condition as far as color, maturity, size, and firmness since they were part of the same batch.

The experimental units are randomly assigned to treatments, resulting in a completely randomized design. We utilized 18 experimental units for one replicate, six in the LDP bag and the rest equally distributed among all other four treatments. Each treatment was performed in three replicates.

### 2.2 Isochoric system

**Figure 1:**
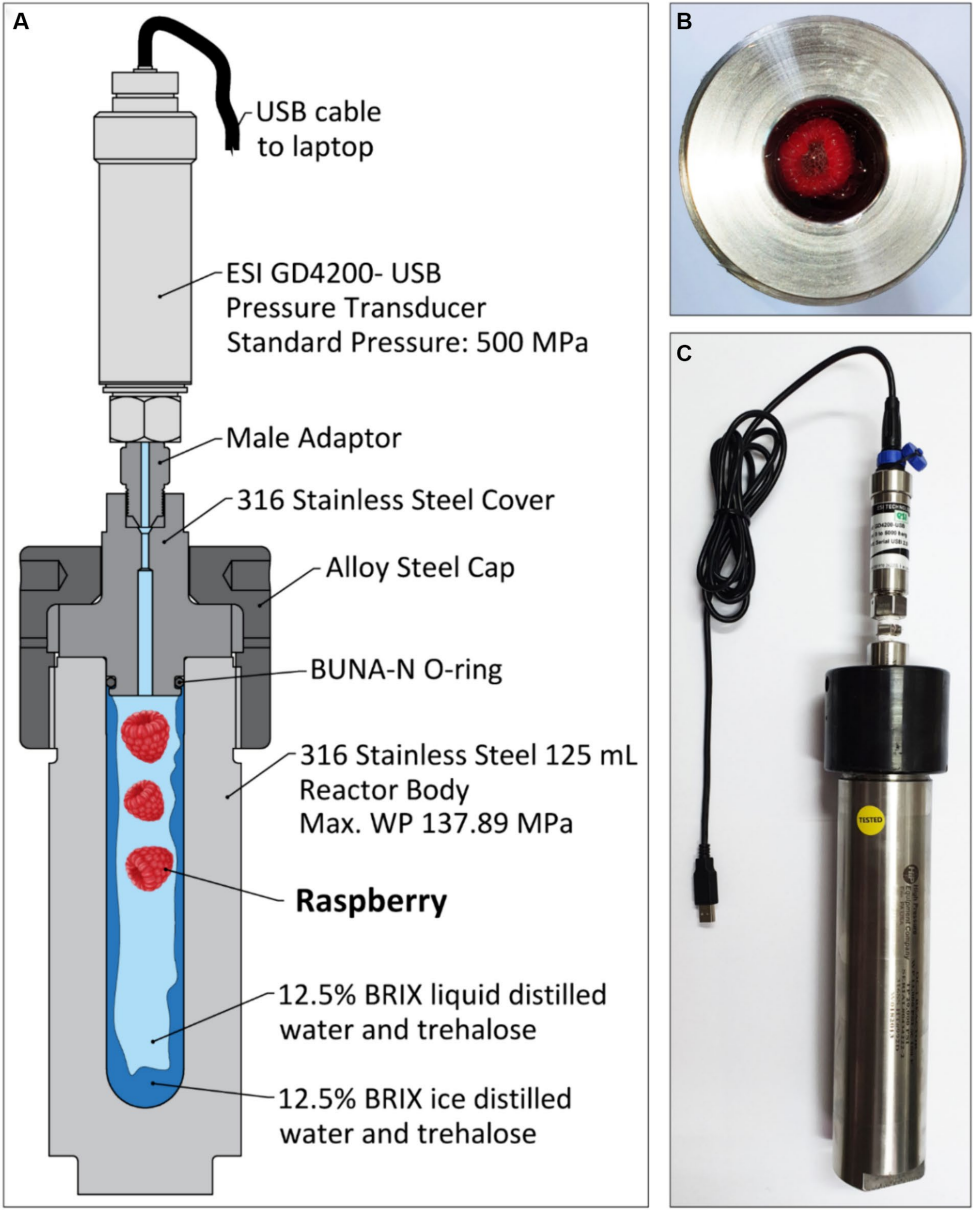
**(A)** Schematic of freezing for preservation of raspberry in an isochoric reactor at equilibrium. **(B)** Top view of the isochoric reactor with the raspberries inside; **(C)** Photograph of the isochoric system, consisting of the high-pressure reactor, the pressure transducer, and the USB cable for connection to the laptop.

The isochoric system consists of an OC-1 type high-pressure vessel from High Pressure Equipment Company (Erie, PA, United States) and a pressure transducer manufactured by ESI Technology GD4200-USB in the United Kingdom for a range of 0 to 72,519 PSI, or 0 to 500 MPa, used to measure the inside pressure. The high-pressure reactor, as can be observed in Figure 1, is made of 316 stainless steel, has an internal volume of 125 mL, and has an internal diameter of 1″. The outside diameter of the reactor is 3″ 1/5 and the height is 6″. It is designed for a maximum working pressure of 20,000 PSI, or 137.89 MPa. The reactor has a 316 stainless steel cover equipped with a Buna-N elastomer O-ring, suitable for applications with negative temperatures down to −40°C. The O-ring provides good sealing between the reactor and the cover. An alloy steel cap threaded onto the isochoric reactor is used to close the reactor.

This study uses the devices and techniques also used in the references (28, 29). For simplicity, the system and procedures are also described here.

### 2.3 Experimental setup—equipment

**Figure 2:**
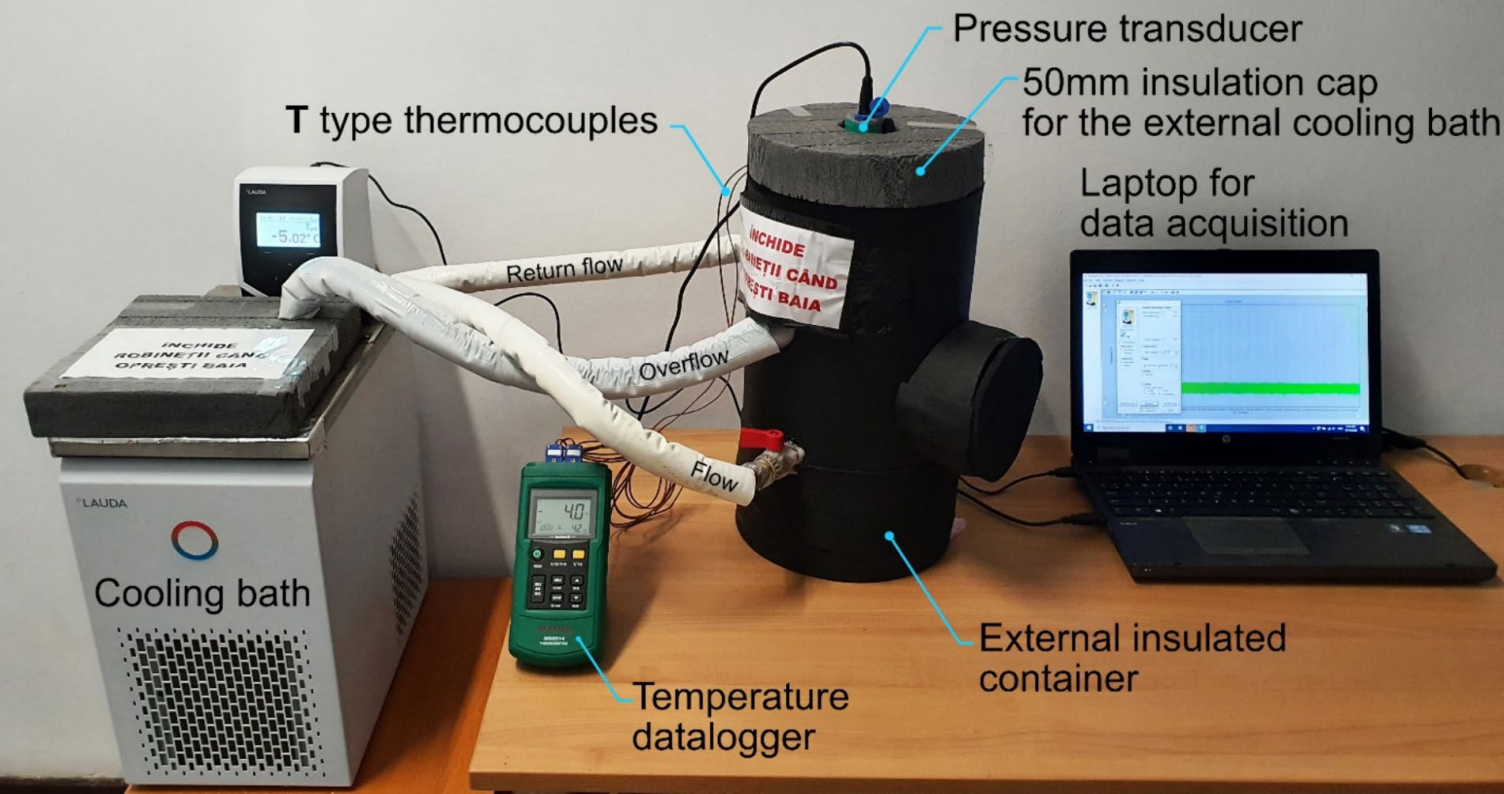
Photograph of the raspberry isochoric preservation experiments setup.

In the current experiment, we used the isochoric chamber previously presented, connected to a pressure transducer, to measure the inside pressure. We connected this pressure transducer via ESI-USB Dynamic software to a laptop (ProBook 66570b, Hewlett Packard Enterprise, India), on which we visualize, store, and export data. The temperature is measured using two PerfectPrime TL0024 T-type thermocouples (2 m long, specific for high accuracy measurements in the refrigeration and cryogenics field, with excellent repeatability between −200°C and 260°C), connected to a digital thermometer (MS6514 Mastech Digital Inc., China), which is connected to the same laptop to display and record temperatures. To limit the effects of ambient temperature, we used polyethylene insulation on the tubes attached with electrical tape to cover the pressure transducer. For safety reasons, we recommend using a safety head with a rupture disk at pressures of 120 MPa, which we have not used because of our vast experience working with these devices. To perform the measurements, the assembly described above is immersed in a cooling bath that uses ethylene glycol recommended for temperatures down to −30°C. The temperature is controlled by a cooling device (RE 1225 S, LAUDA DR. R. WOBSER GMBH & CO. KG, Germany), which can lower the temperature to −25°C within the time and temperature set in the experimental protocol. The cooling bath is connected to an externally insulated container that can provide the necessary volume for the isochoric system. The externally insulated container has an internal volume of 50 liters, a diameter of 190 mm, a height of 400 mm, and is made of PVC. The bath is insulated with a 9-mm elastomer self-adhesive insulation sheet. We used an externally insulated container because the commercial cooling bath has an internal depth of only 200 mm, and our system needs at least 350 mm. The experimental setup with all the components is presented in Figure 2.

### 2.4 The experimental protocol

We measured the brix of 10 fruits from the lot and found an average of 12.5% brix. For the experiments, we made a 12.5% Brix solution made of trehalose powder (SOSA Ingredients S.L., Spain) and steam-distilled water (European Drinks SA, Romania). The isochoric system was filled with the trehalose solution; we added three raspberry fruits inside, and then we sealed the reactor. A trehalose solution concentration of 12.5% Brix was in osmotic equilibrium with raspberries, as no mass change was observed after raspberries were kept in this solution at 5°C for 1 day. Five different methods to preserve fresh raspberry for 7 days: at room temperature (+21°C) and 44% RH (laboratory conditions); in the fridge, at +3°C and 65% RH; in the freezer, at −21°C and 100% RH; isobaric at −4°C in a plastic bag, immersed in the cooling bath; and isochoric at −4°C in the isochoric system. For the isobaric preservation method in the LDPE plastic bag, we used six raspberry fruits, and for the rest of the preservation methods, we used three raspberry fruits in each experiment. For the fruits preserved in the LDPE plastic bag, we used the same 12.5% Brix trehalose solution.

After the isochoric reactor was sealed and closed, it was fully immersed in a recirculating cooling bath at −4°C. The temperature difference between the cooling bath and the externally insulated container was 1°C for the entire duration of the experiment. This way, for the isochoric preservation and isobaric preservation in the LDPE bag methods, the temperature was −4°C. The freezing temperature was chosen based on preliminary data (30, 31). Temperatures near the freezing point of water are recommended for the storage and distribution of raspberries. Based on this information and considering raw raspberries are 84–90% water (32), At ripeness, we chose a temperature of −4°C, just below the freezing point of water. Lower freezing temperatures result in higher pressures in the isochoric reactor. The average pressure at the preservation temperature of −4°C was 43.8 MPa. At the end of each experiment, the cooling bath was manually set to 20°C, but at 5°C, the reactor was removed from the cooling bath and placed in a room-temperature water bath for 10 min before opening.

### 2.5 Weight loss

For all storage methods, raspberries with initial masses of 2.4–4.2 g were used to measure mass changes. The mass change of raspberries was determined gravimetrically using a Digi weight MH-200 pocket scale (MH-200, Guangzhou Juheng Electronic Co., Ltd., China), with a scale range from 0.01 to 200 g.

### 2.6 Microscopy

All samples in this study were microscopically examined to observe the microstructural changes inside the fruits caused by each preservation method. Raspberries from each group were cut with a scalpel parallel to the longitudinal axis. The fresh raspberries and the fresh raspberries immersed in the liquid nitrogen samples were analyzed at the beginning of the experiment, on the first day of processing. The fruits preserved in the isochoric system, in the LDPE bag, in the fridge, and in the freezer were analyzed after 7 days of preservation. The samples were observed with a digital microscope with incident and direct LED illumination (Bresser 5,201,010, Bresser GmbH, Germany), using the 10X objective. All the images were viewed on the LCD screen of the microscope and then stored on its 32 GB SD card as JPG format, with a resolution of 3,072 × 1728 px. The images obtained were processed and analyzed with the scientific imaging software Image J, version 1.53 t.

### 2.7 Brix percentage

The sugar quantity in the processed fruits was measured with a portable refractometer (0–90%, Milton Industries Inc., China). The higher the brix value, the sweeter and tastier the fruits are. We calibrated the instrument with distilled water. After each measurement we cleaned the measured solution on the surface of the prism and daylight plate with distilled water and after wiped with a moist cotton cloth.

### 2.8 pH measurement

To measure the pH of the fruits, we used a portable pH meter from Testo (Testo 206-pH1, Testo, RO). The pH electrode has a measurement domain of 0 to 14 pH and a precision of 0.02 pH at a resolution of 0.01 pH. To measure raspberry pH, the fruits were minced, cooled to 20°C, and then measured with a pH meter. After each measurement, we cleaned the measured solution from the surface of the pH electrode using distilled water.

### 2.9 Statistical analysis of data

The data presented in this study are average values ± standard deviations. All preservation techniques underwent an analysis of variance, and Tukey’s comparison test was used to determine the significance of any differences between treatments. At *p*-values≤0.05, significance values were assessed. For comparisons between treatment means parameters in the experimental design, Tukey’s pairwise comparison test was used. For each parameter, analysis of variance (ANOVA) was carried out at a 5% threshold of significance (significance levels were tested at *p* ≤ 0.05). Data is presented as mean values ± standard deviation. For all statistical investigations, Minitab version 19 Statistical software was used (Minitab Inc., State College, PA).

## 3 Results and discussions

The isovolumetric storage technique is very simple, consisting only of a high-pressure chamber, a pressure transducer, and a cooling device. Figure 1A shows a schematic of the isochoric device with all the components that were used in our experiments. Control over the isochoric preservation is done either by pressure or temperature to completely specify the system. As can be observed in Figure 1A, in an isochoric system at equilibrium, we have the inside solution in a two-phase state of liquid and ice in a closed, fixed volume. In Figures 1B,C, two photographs of the isochoric device are shown, from the top and from the side. It is a 316 stainless steel commercial pressure vessel covered with a cylinder made from the same material and then capped by an alloy cap.

**Figure 3:**
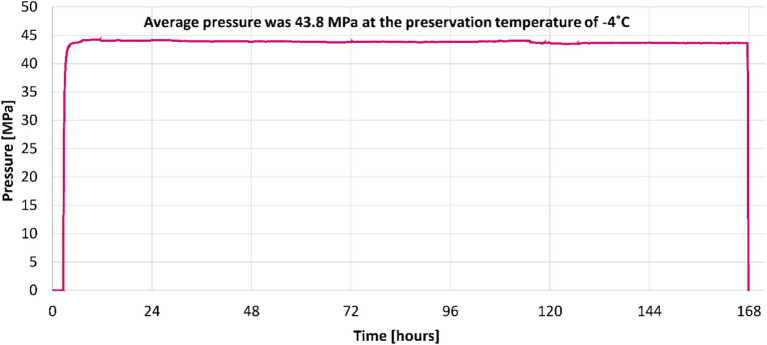
This figure shows the pressure measurement over time (7 days) during the raspberry preservation experiment in the isochoric system. The pressure in the isochoric chamber was measured during the experiment using the pressure transducer in Figure 1.

In Figure 2, the complete isochoric preservation setup is presented. Besides the isochoric reactor and the pressure transducer, it can be observed that for cooling the system, we used a cooling bath, and to analyze and store the temperature and pressure data, we used a laptop. Figure 3 was obtained from measurements made during a 7-day isovolumic raspberry storage experiment using the pressure transducer in Figure 1. It shows a typical curve representing the pressure change over time during the isochoric cooling process in experiments down to a temperature of −4°C. An interesting point is that the pressure reaches a steady state and remains at that value for 7 days until the end of the experiment. This indicates that the isovolumic system has reached thermodynamic equilibrium. The time to attain equilibrium manifestly depends upon the thermal mass of the recipient and the heat transfer coefficient to the cooling bath. The average pressure at equilibrium was 43.8 MPa. In all experiments, the samples reached isochoric thermodynamic equilibrium, and the results represent the state of the treated material after reaching thermodynamic equilibrium.

**Figure 4:**
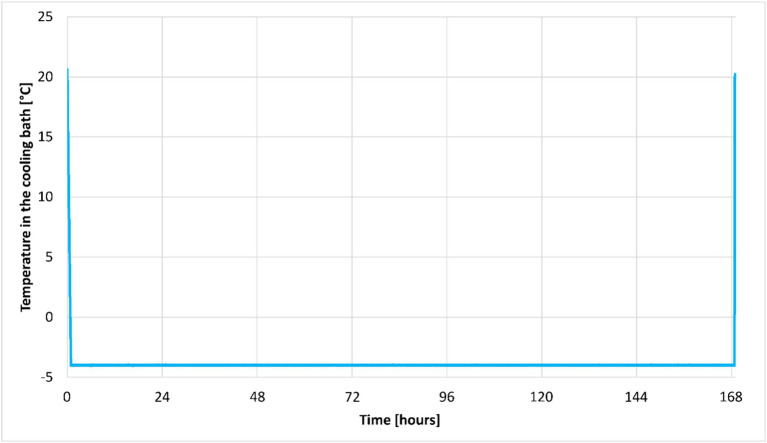
This figure shows the temperature plot over time (7 days) during the raspberry preservation experiment in the isochoric system.

Figure 4 was derived from measurements taken during a seven-day isochoric raspberry storage experiment using the temperature datalogger in Figure 2. It shows a typical curve showing the temperature change over time during the isochoric cooling process for experiments down to a temperature of −4°C. Basically, we lower the cooling bath until the preservation temperature is reached, after which, at the end of the experiment, we bring the bath back to the initial temperature.

### 3.1 Weight loss

Weight loss after preservation is a major interest in the food industry. It occurs during storage of any food product, including raspberries.

**Figure 5:**
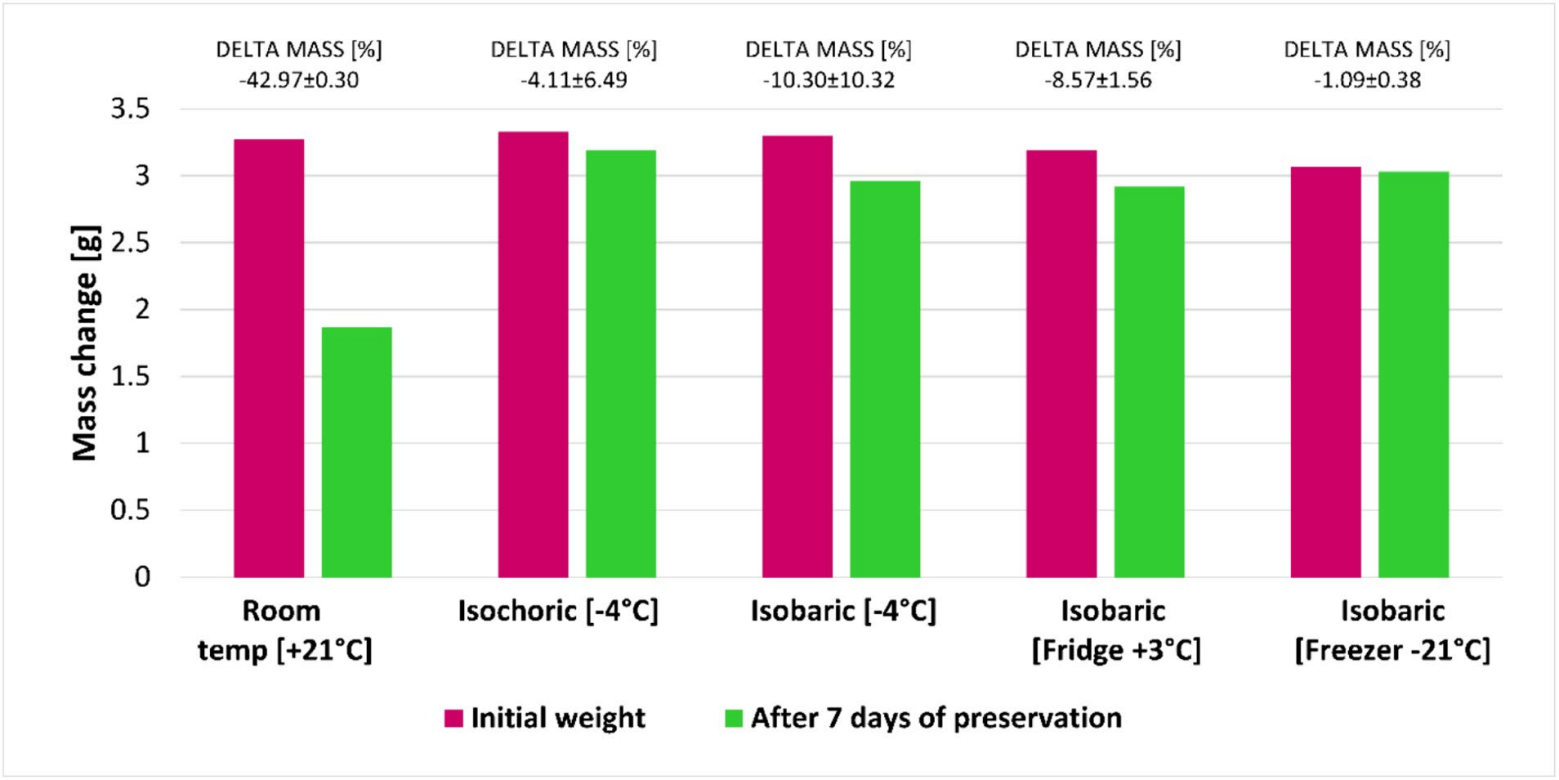
Average mass change of the raspberries after 7 days of preservation with five different methods. The average mass change was determined by analyzing the weight of the samples before and after the storage process. Water present on the surface of the samples was absorbed by filter paper before weighing. Three samples per replicate were measured for weight loss for each storage method.

Figure 5 compares the weight loss before and after 7 days of preservation with five different methods described in the experimental protocol. This figure proves that there is no statistically significant change in weight in any of the four preservation methods that involved negative preservation (one close to zero degrees), in contrast with room temperature preservation. Keeping the fruits at room temperature in isobaric is not a good option since the weight loss was at an average of 42% or more. The weight loss observed in this study is consistent with the findings from many other analyses (22, 23, 33). As far as we know, the fact that there is little or no weight loss after isochoric preservation of raspberries at temperatures below the freezing point of water is new for the food preservation field.

### 3.2 Macroscopic appearance

**Figure 6:**
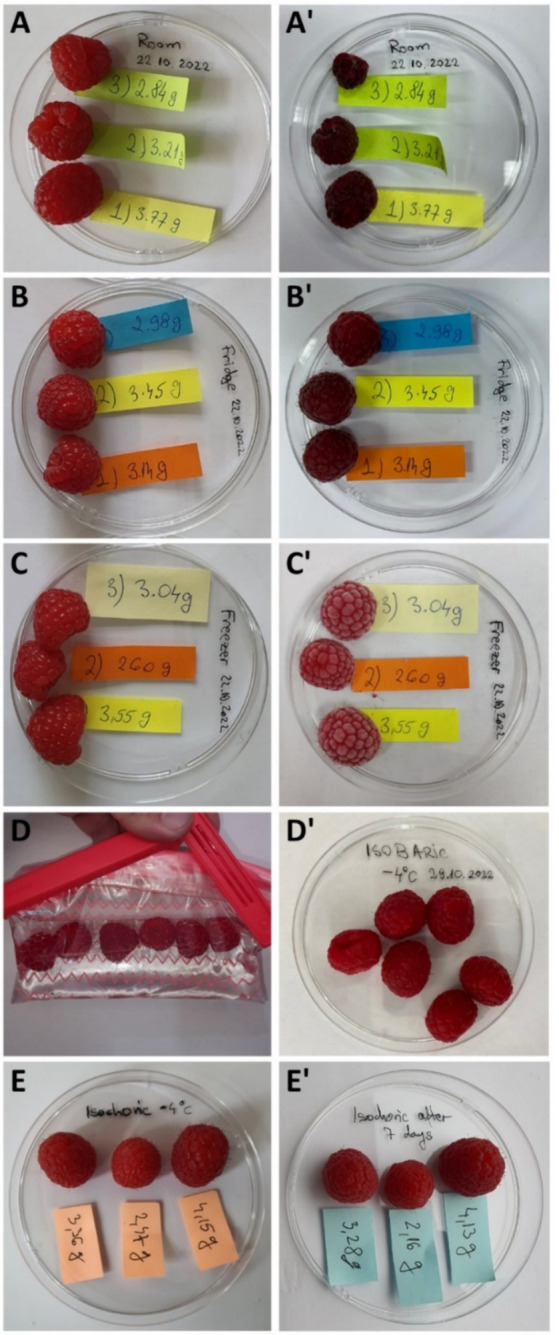
Photographs of the raspberries used in the experiments Fresh fruits are on the left side: the samples for preservation at room temperature **(A)**, the samples to be preserved in the fridge **(B)**, the samples to be preserved in the freezer **(C)**, the samples to be preserved in isobaric conditions in isotonic trehalose solution in a plastic bag **(D)**, and the samples to be preserved in the isochoric system in the same isotonic solution **(E)**. The photographs from the right side **(A’–E’)** present the same samples after 7 days of preservation with the methods described earlier.

In Figure 6, the macroscopic appearance and color of the fruits can be observed after 1 week of preservation with five methods, compared with the same fresh fruits before preservation. Appearance and color are the first important attributes that determine whether consumers will accept or reject a product. Consequently, these are some of the most remarkable attributes in the fruit industry (34–37). As can be observed from the fruits preserved at room temperature and in the fridge, after 7 days their color is much darker than that of the fresh fruits. The fruits from the freezer (Figure 6C’), a few seconds after removing them from the freezer, have a dull color or frosty look, covered with specific, white-colored ice. The fruits preserved isobarically in the plastic bag at −4°C and isochorically at −4°C look the same as the fresh fruits but tend to have a darker color. This might be because of the trehalose solution, which could influence the amount of anthocyanin accumulation during storage.

### 3.3 Microscopic comparison

**Figure 7:**
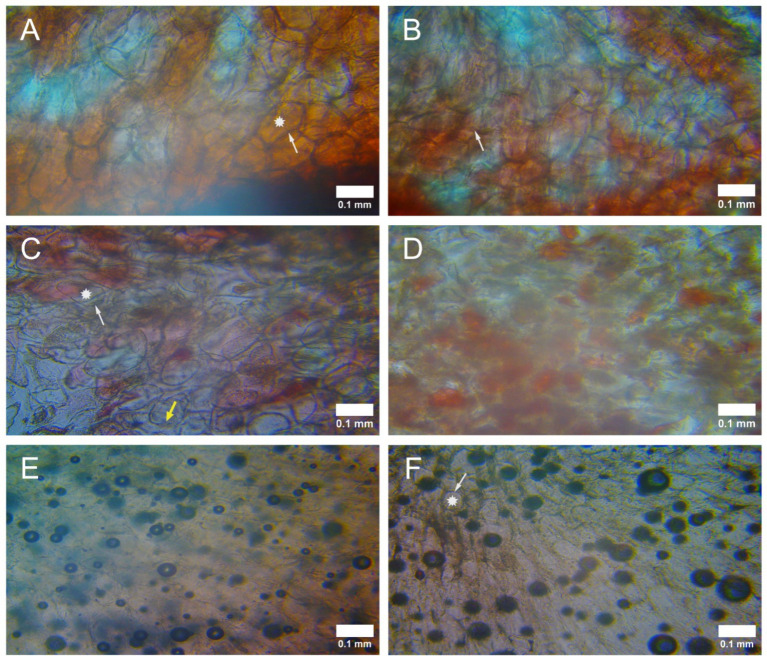
Comparison between fresh raspberry tissue **(A)**, raspberry tissue after 7 days of isobaric preservation in the fridge **(B)**, raspberry tissue after 7 days of isochoric preservation **(C)**, raspberry tissue after 7 days of isobaric preservation at −4°C **(D)**, tissue of raspberry after 7 days in the freezer **(E)**, and tissue of fresh raspberry in liquid nitrogen **(F)**. A raspberry cell is marked by a star. The cells are connected by a membrane, pointed by a white dashed arrow. Plasma membranes had ruptured, as pointed by a yellow arrow. Dimensions are given by the scale bar.

Figure 7 shows microscopic images of all treated samples and the effect of isovolumic preservation on the raspberry tissue. Photomicrographs show the appearance of the samples at 10x magnification for the fresh raspberry (Figure 7A), after 1 week of isobaric preservation in the fridge at +3°C (Figure 7B), after 1 week of isochoric preservation at −4°C (Figure 7C), after 1 week of preservation in a LDPE bag filled with 12.5% trehalose solution at −4°C (Figure 7D), after 7 days of preservation in the freezer (Figure 7E), and for the fresh raspberries immersed in liquid nitrogen (Figure 7F). The arrow in the figure points to the cell membrane, while the star indicates a cell. It is evident that the cell wall of fresh and isochoric preserved fruits is intact and surrounds the cells. In contrast, in the fruits immersed in liquid nitrogen and preserved in the freezer, the cell walls are impaired, less in the fruits immersed in liquid nitrogen and greater in the fruits preserved in the freezer. Furthermore, it can be observed that the fruits preserved in the isochoric system for 7 days have the same reddish appearance at the microscope, while in the other two cases of cold preservation, this appearance is missing. Though the cells in the isochorically preserved fruits show a distance between them, we might say the cell walls thickened. Thicker cell walls than in fresh fruits and some of the cells being colorless indicate plasma membranes had ruptured and anthocyanin leakage had occurred (Figure 7C). Anthocyanins and carotenoids are considered as the primary pigments responsible for the coloration of raspberries (38). Another interesting observation is that after preservation in the isochoric system, there are no traces of mechanical destruction of the tissue due to the presence of ice. In contrast, in the other two methods, the black circles scattered over the tissue indicate the presence of ice crystals and thus mechanical damage to the tissue. The mechanism of damage during isobaric freezing with conventional methods was discussed in the first section of the study. In an isochoric system, only a fraction of the fluid inside the reactor is in the solid state, while the preservation of biological matter takes place in the liquid state. As presented in Figure 1A, the ice in the isochoric systems is produced mostly on the bottom of the reactor or on the walls. Ice is therefore formed away from biological material, and preserved biological material is safe from ice damage. Thus, freezing-induced cell damage mechanisms are eliminated.

### 3.4 Effect of freezing on brix (%)

The brix percentage did not change significantly due to freezing, regardless of the preservation method. In fact, it seems that the presence of cold causes the sugar level in the fruit to drop. In the case of fruits left at room temperature, it can be observed that the sugar level has increased significantly, reaching almost 37% on average. This leads us to the conclusion that the presence of cold slows down the metabolic activity at the cellular level, which allows the preservation of the fruits. The percentage (weight) of sugar in the raspberry juice is equivalent to a brix degree (°Bx) when measured at 20°C. In the case of isochoric, the average decrease (13.51%) in Brix percentage was lower than that in the case of the fruits preserved isobaric at −4°C in the fridge. The smallest decrease in Brix percentage (11.77%) was in the case of the fruits preserved in the freezer at −21°C.

### 3.5 Effect of freezing on pH

**Figure 8:**
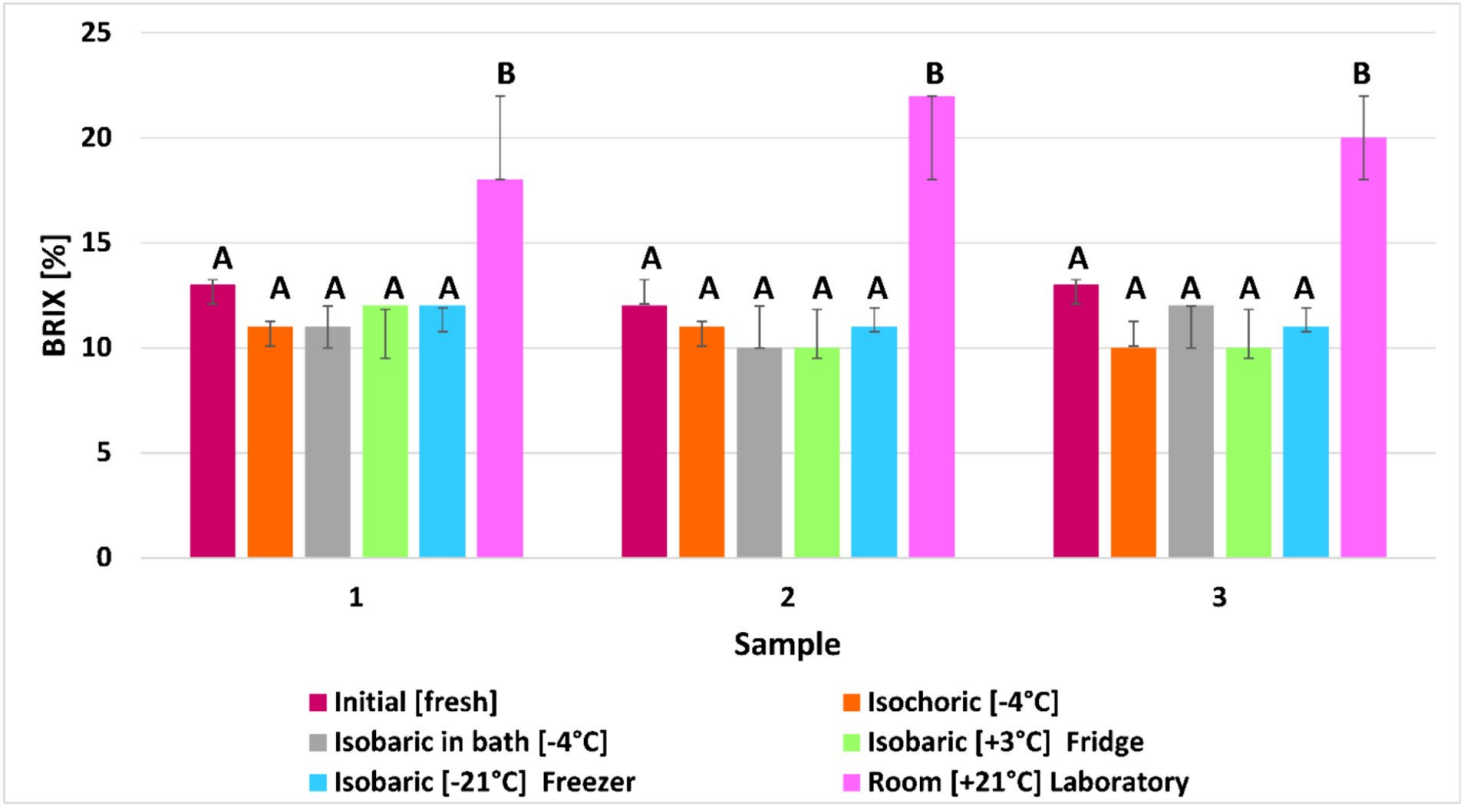
Effect of freezing under five different methods on Brix value (%) The same letter indicates no suggestive difference between treatments at a 95% confidence interval (no statistical difference at *p* ≤ 0.05). The error bars show the standard deviation of each treatment’s samples.

Changes in pH during freezing of raspberries under isochoric, isobaric, and isobaric refrigeration compared with initial values and the fruits kept at room temperature for 7 days are shown in Figure 8. Further experimental information is required to establish the exact nature of the relation between pH and the psychochemical changes in the frozen and unfrozen phases of raspberries and their quality. In the case of isochoric, the average decrease (18.75%) in pH percentage was similar to that in the case of the fruits preserved isobaric in the LDPE bag filled with 12.5% trehalose solution. The smallest decrease in pH percentage (7.64%) was in the case of the fruits preserved in the fridge at +3°C.

**Figure 9:**
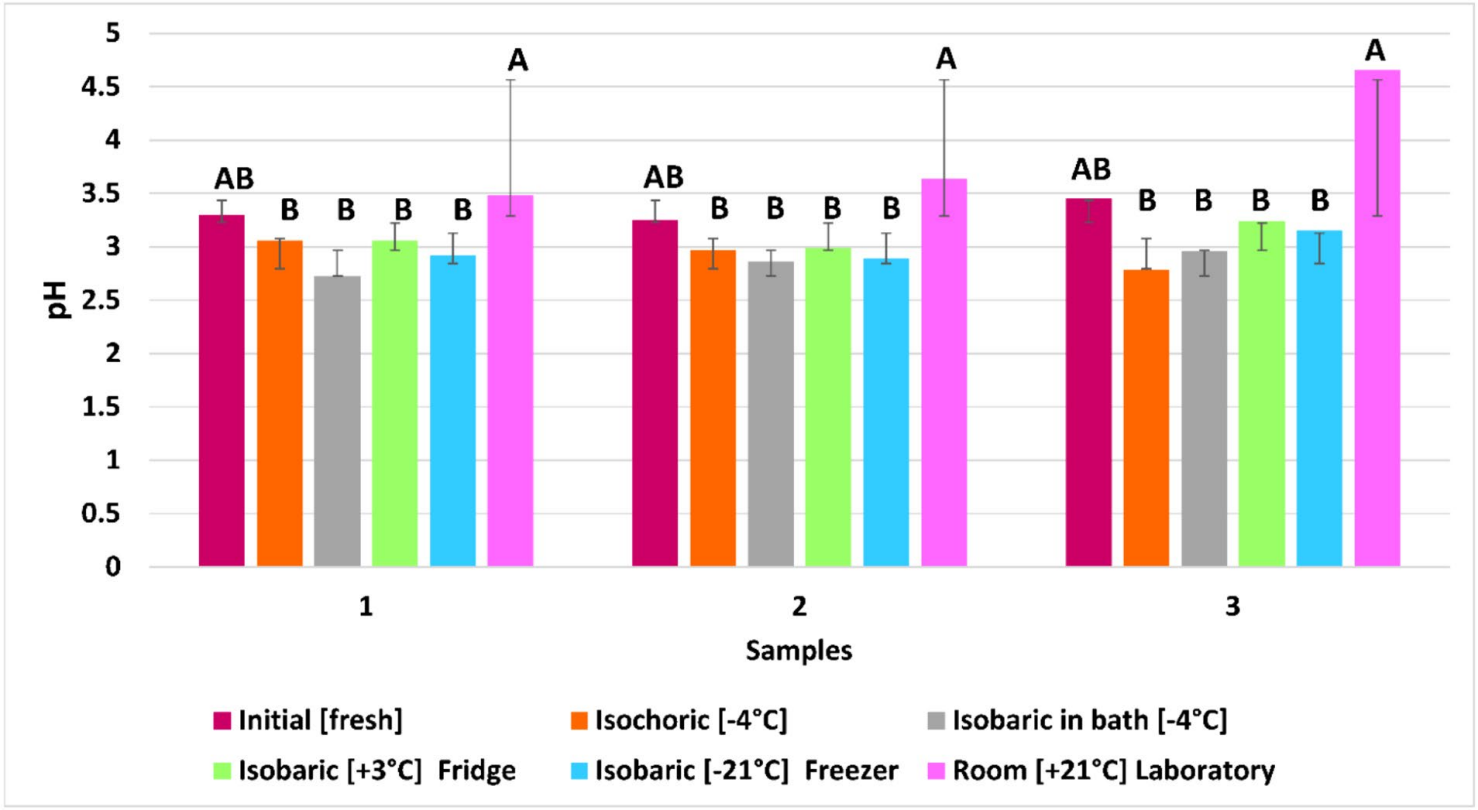
Effect of freezing under five different conditions on pH value. The same letter indicates no suggestive difference between treatments at a 95% confidence interval (no statistical difference at *p* ≤ 0.05). The error bars show the standard deviation of each treatment’s samples.

Changes in pH in raspberries during freezing and preservation are influenced by enzymatic activity, metabolic reactions, microbial growth, and the effects of freezing and thawing. Enzymes like polyphenol oxidase cause browning and pH shifts, while respiration and fermentation produce organic acids that lower pH. Microbial activity and cell rupture during freezing release organic acids, altering solute concentration and pH. Chemical reactions such as the Maillard reaction and oxidation further impact pH. These changes affect fruit quality, influencing flavor, color, texture, and nutritional value. Lower pH can lead to sour flavors, color degradation, enzymatic browning, and changes in firmness. Understanding these mechanisms and their effects on pH can help optimize preservation techniques, such as comparing isochoric and isobaric freezing methods, to maintain raspberry quality (Figure 9).

## 4 Conclusion

Storage under isochoric freezing preserved the raspberry macroscopic texture and improved overall quality compared to fruits frozen under isobaric conditions. The effectiveness of isochoric freezing was attributed to the absence of ice crystals in the fruit during storage, resulting in reduced cell damage. The metabolic reactions in the fruits might have been delayed and the chemical reactions minimized. In contrast, freezing at atmospheric pressure destroyed the cells through ice formation. Raspberries stored at 4°C in an isochoric system exhibited desirable properties in terms of mass, macroscopic and microscopic appearance, color, and texture. This study provides the first practical demonstration of raspberry preservation using the isochoric freezing method. However, future studies should focus on the nutritional qualities, sensory quality, and microbial safety of the raspberries. Another challenging assessment is the analytical firmness of the raspberries due to fruit morphology heterogeneity, a lack of standardized methodologies, and resources available only for a few varieties (39). In summary, storage under isochoric freezing can extend the shelf life of raspberries, resulting in higher-quality fruit and consequently less fruit waste.

Future research should focus on enzymatic activity control, impact of microbial growth, cellular mechanisms of pH change, comparison of freezing techniques, long-term storage effects, nutritional value assessment, consumer acceptance studies to optimize raspberry preservation and maintain fruit quality.

## Data availability statement

The raw data supporting the conclusions of this article will be made available by the authors, without undue reservation.

## Author contributions

SC: Conceptualization, Data curation, Investigation, Methodology, Software, Writing – original draft. GB: Conceptualization, Data curation, Investigation, Methodology, Software, Writing – original draft. BV: Data curation, Investigation, Visualization, Writing – original draft. MT: Data curation, Investigation, Visualization, Writing – original draft. GN: Conceptualization, Funding acquisition, Project administration, Resources, Supervision, Validation, Writing – review & editing.
